# Versatile Graphene Oxide and Its Organo-Modified Analogs for the Removal of Pharmaceutical Compounds

**DOI:** 10.3390/ma19101916

**Published:** 2026-05-07

**Authors:** Emilie Fragnaud, Louis Hennet, Eric Bourhis, Samuel Guillot, Sandrine Delpeux, Fabrice Muller, Yoshiyuki Sugahara, Régis Guégan

**Affiliations:** 1 Institut des Sciences Chimiques de Rennes, ISC-UMR 6226, CNRS-Université de Rennes, 263 Avenue du Général Leclerc, 35700 Rennes, France; emilie.fragnaud@univ-rennes.fr; 2 Interfaces, Confinement, Matériaux et Nanostructures, ICMN-UMR 7374, CNRS-Université d’Orléans, 3 Avenue de la Recherche Scientifique, 45071 Orléans CEDEX 2, France; eric.bourhis@cnrs-orleans.fr (E.B.); samuel.guillot@cnrs-orleans.fr (S.G.); sandrine.delpeux@cnrs-orleans.fr (S.D.); fabrice.muller@univ-orleans.fr (F.M.); 3 Department of Applied Chemistry, Faculty of Science and Engineering, Waseda University, 3-4-1 Okubo, Shinjuku-ku, Tokyo 169-8555, Japan

**Keywords:** graphene oxide, hybrid materials, adsorption, pharmaceuticals, contamination, water

## Abstract

Adsorption properties of graphene oxide (GO) and its organo-modified analog (GO-HDTMA), treated with the hexadecyltrimethylammonium (HDTMA) cationic surfactant, were evaluated for the removal of persistent pharmaceutical products (PPs): an anionic diclofenac (DCF) anti-inflammatory, a cationic metoprolol (MTP) beta-blocker, and a nonionic sulfamethoxazole (SMX) antibiotic. Adsorption isotherms, fitted by Langmuir and Freundlich models, together with FTIR data, demonstrate that both GO and GO-HDTMA are effective adsorbents for DCF. The adsorption is primarily governed by both π–π and van der Waals interactions, leading to saturation of the accessible C sp^2^ carbon domains at a maximum uptake of 4.2 mmol g^−1^. In contrast, due to its cationic nature, MTP is not removed using GO-HDTMA, while it is effectively adsorbed at about 0.5 mmol g^−1^ for GO via electrostatic attractive forces. SMX is adsorbed by both materials, although its uptake remains limited on GO. The presence of hydrophobic domains in GO-HDTMA enhances SMX adsorption through weak intermolecular interactions. These results highlight the tunability of GO-based hybrid materials and their potential for the selective removal of a large spectrum of emerging pharmaceutical contaminants.

## 1. Introduction

The synthesis of numerous organic compounds for therapeutic purposes has revolutionized the management and treatment of diverse pathologies. However, the widespread use of these compounds has led to their continuous release into the environment. Currently, more than 3000 pharmaceutical products (PPs) are in use worldwide, and a substantial fraction is excreted unmetabolized or as active metabolites, resulting in their accumulation in aquatic environments [1,2,3,4,5,6,7,8,9,10]. Among the most frequently detected compounds are diclofenac (anti-inflammatory), sulfamethoxazole (antibiotic), and metoprolol (beta-blocker), which have been reported in both natural and drinking water supplies [1,2,4,6,9,11]. Although advances in analytical techniques, such as gas chromatography and high-performance liquid chromatography coupled with mass spectrometry, have enabled detection at trace levels, these developments have also highlighted the limited efficiency of current water treatment technologies, for which regulatory thresholds are often lacking [1,2,3,6,9,10,12]. Among water remediation strategies, adsorption is widely regarded as an efficient and cost-effective strategy for water treatment, particularly when using porous materials with high specific surface area and abundant active sites. Activated carbons have demonstrated broad applicability; however, their performance is limited for polar compounds and high-molecular-weight species, (>3000 Da), which may clog their micropores [13].

**Figure 1:**
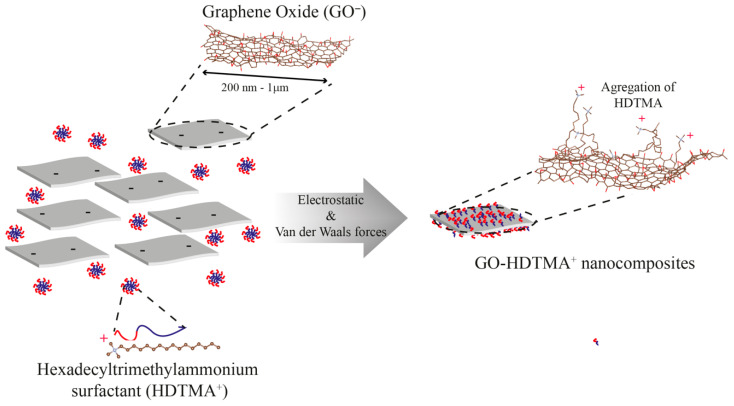
Colloidal association of hexadecyltrimethylammonium (HDTMA) cationic (+) surfactant to anionic (−) graphene oxides through both electrostatic and Van der Waals interactions, leading to their decoration and the formation of GO-HDTMA cationic nanocomposites.

Alternative carbon-based materials such as carbon nanotubes have also been explored, but they suffer from aggregation/flocculation, reducing their accessible surface area. In contrast, graphene oxide (GO), produced by oxidation and exfoliation of graphite, offers a high specific surface area and a rich surface chemistry, including carboxyl, hydroxyl, and epoxy groups, making it a versatile adsorbent [14,15,16,17,18,19,20,21,22,23,24,25,26,27]. Surface functionalization of GO with cationic surfactants provides an effective strategy to tailor its interfacial properties. Such modification can reverse the surface charge and introduce hydrophobic domains, thereby enhancing adsorption of specific classes of pollutants. This approach has been successfully applied to other materials, such as clay minerals, where intercalation of surfactants modifies both interlayer spacing and adsorption behavior [9,23,28,29]. In phyllosilicate materials, cationic surfactants readily exchange with inorganic interlayer cations, leading to an expansion of the interlayer spacing that depends on the loading and organization of the intercalated species. This modification not only creates a hydrophobic environment but also introduces a net positive charge, thereby enhancing the affinity toward anionic pharmaceutical products (PPs) [2,9,24,25,26,27,28,30,31,32,33,34]. Accordingly, organoclay materials have demonstrated high efficiency for the removal of anionic compounds such as diclofenac, as well as other negatively charged species that are poorly retained by unmodified clays [2,9,28]. A similar strategy can be applied to graphene oxide (GO), where adsorption of cationic surfactants introduces positively charged organic moieties and modifies the interfacial properties. In addition, surfactant aggregation on the GO surface can induce a foam-like morphology (Figure 1), increasing the effective interfacial area and thus promoting mass transfer of pollutants from the aqueous phase to the adsorbent [23,29,35].

In this context, this study aims to develop a hybrid adsorbent based on graphene oxide (GO) nanosheets functionalized with the hexadecyltrimethylammonium (HDTMA) cationic surfactant, as well as to characterize its structural, morphological, and colloidal properties using complementary techniques. The environmental performance of the resulting GO-HDTMA material is evaluated through a comparative adsorption study involving three pharmaceutical compounds with distinct charge states: anionic diclofenac, cationic metoprolol, and nonionic sulfamethoxazole. Adsorption capacities are quantified using UV–visible and infrared spectroscopies and systematically compared with those of pristine GO. The experiments are conducted over a broad concentration range, well above environmentally relevant levels, to elucidate the main adsorption mechanisms and to determine the maximum uptake capacities of both GO and GO-HDTMA materials.

## 2. Materials and Methods

Graphite powder (Sigma-Aldrich, St. Louis, MO, USA, 99.9%, <45 μm), sulfuric acid (95%), potassium permanganate (99.3%), hydrogen peroxide (30 wt%), hexadecyltrimethylammonium (HDTMA) cationic surfactant and hydrochloric acid (35–37%) were purchased from Sigma-Aldrich and used as received.

Graphite oxide was synthesized based on the modified Hummer’s method. Sulfuric acid (25 mL) and graphite (0.5 g) were added to a 500 mL beaker and stirred for 30 min in an ice bath. After adding potassium permanganate (5 g), the mixture was stirred for 1 h. Then, the mixture was stirred at 70 °C for 20 min. Then, pure water (200 mL) was added, and 30% hydrogen peroxide was added drop by drop without observing any bubbles. The precipitate (graphite oxide) obtained by centrifugation (3000 rpm, 10 min) was washed by centrifugation with 5% hydrochloric acid and pure water. The precipitate was then dispersed in pure water (50–100 mL), and the dispersion was centrifuged to remove sulfate and other ions that cause a decrease in pH, and the supernatant was discarded. The obtained precipitate was dispersed in pure water and centrifuged to collect the supernatant (8000 rpm, 30 min). Finally, it was concentrated by centrifugation (15,000 rpm, 40 min) to get a colloidal dispersion of graphene oxide at a concentration of about 0.5 g L^−1^.

GO-HDTMA composites were prepared based on the latter GO dispersion collected at 0.5 g L^−1^, which was mixed to HDTMA concentrations of 1.82, 3.64 and 7.2 g L^−1^, corresponding to mass ratios of 3.6, 7.2, and 14.6 (HDTMA/GO), respectively. The suspensions were stirred for 2 h and centrifuged twice at 6000 rpm for 30 min to remove the excess surfactant. Solid GO-HDTMA composites were then deposited on a Si wafer in a solid form after evaporation of a solvent in a drying oven at a temperature of 50 °C for 48 h and were characterized by FTIR and Raman spectroscopies. Some GO and GO-HDTMA composites were dried in an oven at 80 °C for 48 h prior thermal gravimetry experiments. The other collected GO-HDTMA composites were then redispersed in water, and their zeta potentials were evaluated to confirm the proper change of GO surface induced by the presence of HDTMA surfactant at different mass ratio. The same GO-HDTMA dispersions were used as adsorbent matrixes for the batch pharmaceuticals removal experiments.

**Figure 2:**
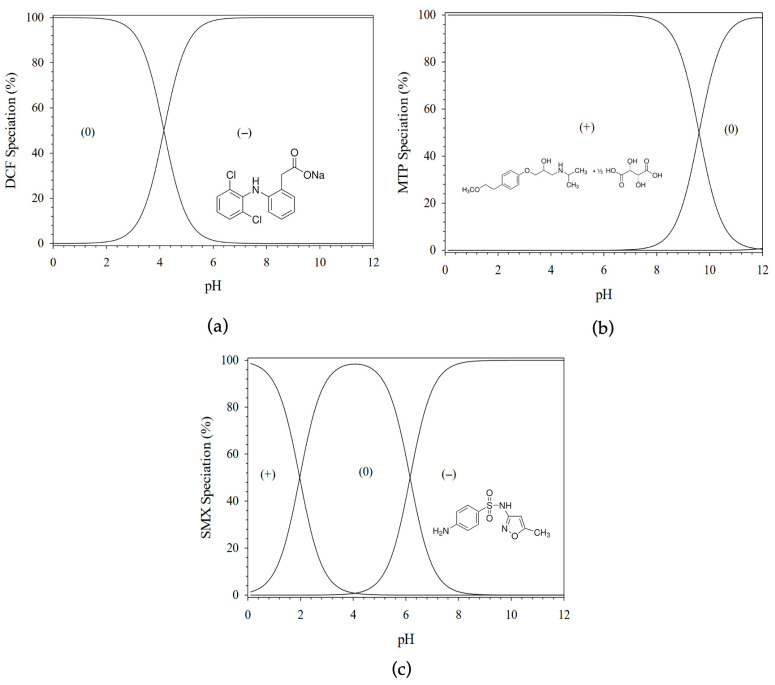
Speciation of the three pharmaceutical products used in this study: (**a**) Diclofenac (DCF), (**b**) metoprolol (MTP) and (**c**) sulfamethoxazole (SMX) mainly anionic (−), cationic (+) and nonionic (0) at a pH = 5, respectively. Data for SMX are adapted from Ref [36].

Diclofenac (2-[(2,6-dichlorophenyl)amino]benzeneacetic acid), purchased from Sigma Aldrich Chemical and assumed to have a purity > 98%, was used in its sodium salt form, which shows a solubility higher than 10 g L^−1^. This pharmaceutical exhibits a log P of approximately 4.51 and a pKa of 4.13. Metoprolol, supplied by Sigma Aldrich Chemical, with an assumed purity of >98%, was used in its tartrate salt form with the molecular formula (C_15_H_25_NO_3_)_2_·C_4_H_6_O_6_. This pharmaceutical shows a good solubility in water (4 g L^−1^ at room temperature) and is characterized by a log P of 1.9, while having a pKa of 9.67. Sulfamethoxazole (4-Amino-N-(5-methyl-3-isoxazolyl)benzenesulfonamide), which was also purchased from Sigma Aldrich Chemical, shows a pKa couple of 6.16 and 1.97 (respectively, for the strongest acidic and basic forms) and has a log P of 0.89 and a water solubility of 610 mg L^−1^ (at 37 °C). At a pH of 5, diclofenac (DCF) is principally in an anionic form (87%) and metoprolol (MTP) is a cationic compound (≈100%), while sulfamethoxazole (SMX) is mainly neutral (90%) (Figure 2).

Batch adsorption experiments were performed to evaluate the uptake of pharmaceutical compounds onto graphene oxide (GO) and GO modified with hexadecyltrimethylammonium (GO-HDTMA). All experiments were conducted in the dark to prevent photodegradation of the pharmaceutical products (PPs) by ambient light, and at a fixed temperature of 20 °C. Batch adsorption experiments of the individual PPs (diclofenac, metoprolol and sulfamethoxazole) onto GO or GO modified by HDTMA were conducted in duplicate using at least 10 initial aqueous solutions ranging from 10 mg L^−1^ to 2 g L^−1^. The solid-to-liquid ratio was kept constant, using 125 mg of the adsorption matrix (GO and its organo-modified analogs) in 250 mL of aqueous PP solution in centrifuge tubes, and the pH was adjusted to a value of 5 by a mixture of HCl acidic and NaOH basic solutions added in the same proportion and volume for all solutions. Samples were shaken on a rotary shaker at 50 rpm for 24 h to reach equilibrium and then centrifuged at 5000 rpm for 25 min. Additionally, the supernatants were filtered through 0.22 µm membranes to remove any remaining solid particles. The collected supernatants after filtration were analyzed by UV–visible spectroscopy. The amount of adsorbed PPs was calculated from the difference between the initial and equilibrium concentrations, allowing determination of the adsorption isotherms. The collected solid sorbents, after being in contact with the three PPs, were dried at 80 °C for 48 h for FTIR and Raman characterizations, for which the samples were deposited on Si wafers.

XPS measurements were performed using an ESCALAB Xi+ X-ray photoelectron spectrometer (Thermo Scientific, Waltham, MA, USA) employing a monochromated Al Kα X-ray source (hν = 1486.6 eV). The C(1 s) level (284.9 eV) was taken as a reference binding energy. High-resolution spectra were collected using an analysis area of 650 µm × 650 µm and a 20 eV pass energy. A charge neutralizer was used during data collection, monitored using the C(1 s) signal corresponding to adventitious carbon. All spectra were collected and fitted using Avantage software (version number 6.6.0; Thermo Scientific), and a smart background was applied. Raman measurements were recorded with a Renishaw spectrometer operating at 514 nm. Images of GO were acquired using a JEOL 2100 transmission electron microscope (JEOL, Tokyo, Japan). Atomic force microscopy (AFM) images were obtained using the AFM multimode function on a Digital Instruments AFM. Thermogravimetric analyses over the temperature range 50–800 °C at a rate of 10 °C min^−1^ under an inert N_2_ atmosphere (flow rate of 150 mL min^−1^) were performed using a Rigaku TG8120 instrument (Tokyo, Japan). Size distributions and corresponding zeta potentials were determined by dynamic light scattering (DLS) using a Zetasizer Nano ZS (Malvern, UK). The concentration of the three PPs before and after contact with GO was determined by UV–visible analysis using an Evolution 220 spectrophotometer (Thermo Scientific). Fourier transform infrared (FTIR) measurements in the range 650–4000 cm^−1^ were recorded using a Thermo Scientific Nicolet 50 FT spectrometer (Waltham, MA, USA) in ATR mode with a diamond crystal and equipped with a deuterated triglycine sulfate (DTGS) detector.

## 3. Results and Discussion

**Figure 3:**
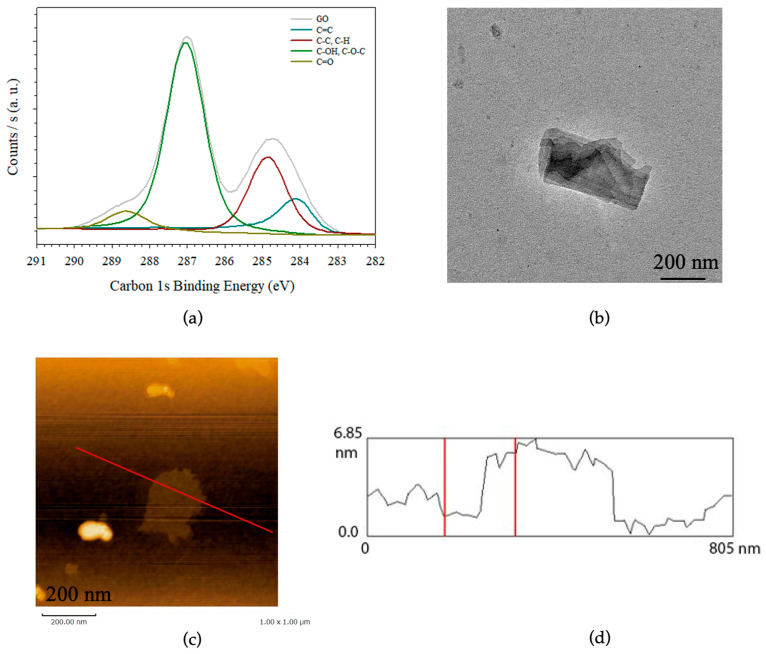
(**a**) Characterization of collected graphene oxide deposited on Si substrate by XPS; (**b**) transmission electron microscopy observations of the graphene oxide deposited in a similar way onto TEM grid; (**c**) atomic force microscopy of the same GO deposition on a Si substrate and (**d**) with the corresponding section analysis.

**Figure 4:**
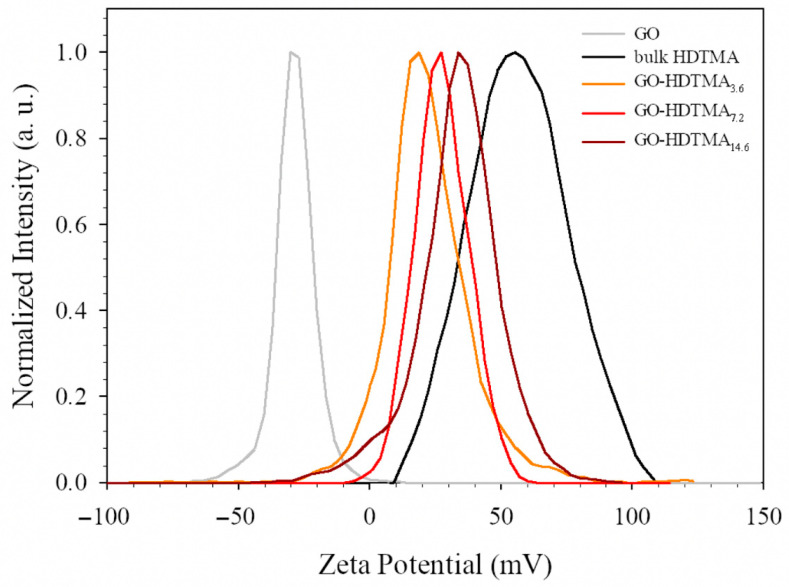
Zeta potential distributions of GO (gray), HDTMA (black) and GO-HDTMA at different mass ratio between the two components HDTMA/GO of 3.6 (orange), 7.2 (red) and 14.6 (dark red).

The Hummer’s modified synthesis method involves the oxidation and exfoliation of a graphite precursor leading to its delamination to carbon nanosheets incorporating oxygen moieties as well as structural defects. These features were characterized by X-ray photoelectron spectroscopy (XPS) (Figure 3), Raman spectroscopy, and Fourier transform infrared (FTIR) spectroscopy (Figure 4). XPS is a widely used technique for both qualitative and quantitative analysis of chemical bonding environments, enabling determination of the oxidation degree of graphene oxide (GO). The XPS spectrum of GO deposited from a colloidal dispersion onto a Si substrate reveals a dominant contribution from C–O and C=O bonds, alongside residual C–C bonds associated with sp^2^-hybridized carbon graphitic domains. Deconvolution of the C 1 s signal indicates that C–O–C and C–OH groups account for 57.5% of the total intensity, while C=O contributes 5.5%. In contrast, C=C/C–C and C–H components represent 13.5% and 23.4%, respectively (Figure 3a and Supporting Information Table S1). The resulting O/C atomic ratio (36.8%) is consistent with those of previous characterizations of GO in the literature, although it indicates a slightly higher oxidation level in the present study. As numerous studies have pointed out, both the morphology and particle size of the graphite precursor strongly influence the reactivity and oxygen groups of the synthesized GO. In this study, the use of a graphite precursor in powder form with small particle sizes (<45 μm) (Figure 3c) likely enhances its reactivity, leading to a higher oxidation degree in the resulting GO and its aggregates [16,17,18,21,35].

Transmission electron microscopy (TEM) measurements, together with atomic force microscopy (AFM) observations, revealed the relatively small lateral size of the synthesized graphene oxide (GO), ranging from only 200 to 300 nm. This value significantly differs from those reported in previous studies and highlights the significant role of the graphite precursor used in GO synthesis. Indeed, GO sheets with large lateral dimensions (~10 μm) can be readily obtained from graphite with large particle or crystallite sizes, and our group has previously demonstrated the reduction of GO size through cavitation induced by high-power ultrasonication. In the present study, the reduced size of the collected GO and its aggregates can be mainly attributed to the characteristics of the graphite precursor, whose particle or crystallite size was below 45 μm. Furthermore, TEM observations at different magnifications suggest a stacking of multiple nanosheets, for which precise determination remains challenging using electron microscopy alone (Figure 3b and Figure S1). In contrast, AFM analyses of GO deposited on a Si substrate enables a more accurate evaluation (Figure 3c and Figure S2). The corresponding height profiles (Figure 3d) indicate an average thickness of 4–5 nm for the GO aggregates. These results suggest that, under the present experimental conditions and using a short-size graphite precursor, GO was not obtained as isolated nanosheets but rather as aggregates consisting of stacks of approximately 4–5 layers. Although dynamic light scattering (DLS) is a valuable technique for estimating the hydrodynamic size of spherical particles, it is not suitable for characterizing 2D nanosheets or non-spherical morphologies. Therefore, as emphasized in previous studies, despite the potential formation of aggregates during deposition and drying on substrates, TEM and AFM remain the most reliable and robust techniques for determining both the lateral size and degree of exfoliation of nanosheets, as well as for assessing their stacking and aggregation behavior.

**Figure 5:**
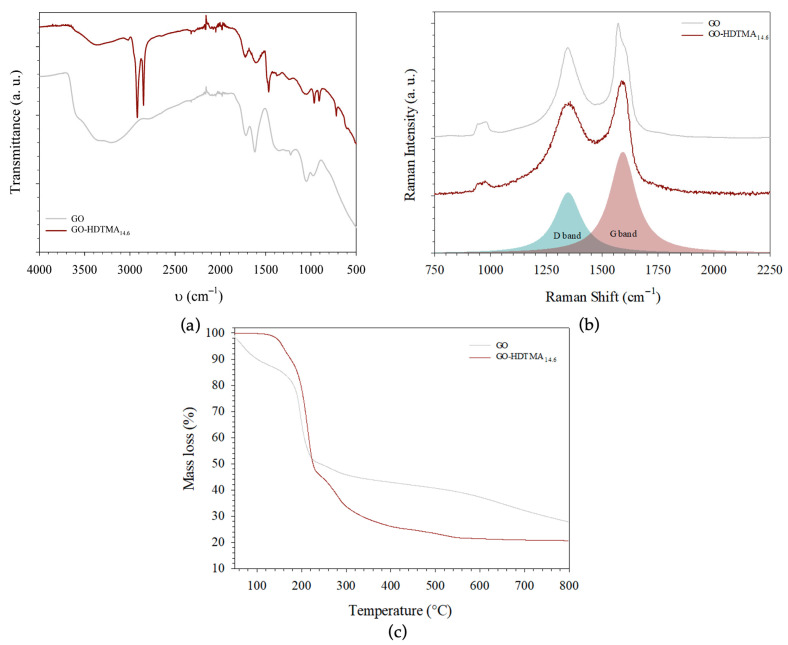
(**a**) Raman scattering of both GO and GO-HDTMA_14.6_ used in this study. (**b**) FTIR spectroscopy measurements of the same materials. (**c**) TGA of both GO and GO-HDTMA_14.6_. The gray and red lines represent the experimental data of GO and GO-HDTMA_14.6_.

The zeta potentials of bulk GO aqueous dispersions, HDTMA solutions, and GO-HDTMA dispersions were measured (Figure 4). As expected, due to the presence of negatively charged oxygen-containing functional groups on the GO surface, the zeta potential of the GO dispersion (0.5 g L^−1^) is −35.3 ± 8 mV. In contrast, the bulk cationic surfactant, at a concentration of 7.2 g L^−1^, forms positively charged spherical micelles with a zeta potential of +60 ± 20 mV. To evaluate the effect of hexadecyltrimethylammonium (HDTMA) adsorption on surface charge, three GO-HDTMA dispersions were prepared with HDTMA concentrations ranging from 1.82 to 7.2 g L^−1^. The suspensions were stirred for 2 h and centrifuged twice at 6000 rpm for 30 min to remove excess surfactant. After collecting GO-HDTMA composites and dispersing them in water, the resulting GO-HDTMA dispersions exhibit zeta potentials of +19, +31, and +37 ± 14 mV for GO-HDTMA_3.6_, GO-HDTMA_7.2_, and GO-HDTMA_14.6_, respectively (corresponding to initial HDTMA concentrations of 1.82, 3.64 and 7.2 g L^−1^). The zeta potential distributions display a single peak, indicating that, in aqueous medium, the majority of the positively charged HDTMA species are adsorbed onto the GO surface, effectively reversing the surface charge from negative to positive [23,29,35]. All GO and GO-HDTMA colloidal dispersions exhibit a Tyndall effect, confirming good particle dispersion in solution; however, their stability is limited to approximately one week, which remains sufficient for pharmaceutical or other water contaminants adsorption experiments. In contrast to our previous studies on large GO, where long-term stable dispersions and liquid crystalline behavior (evidenced by birefringence observations) were observed over months or even years, the reduced stability of the present short-size GO dispersions may be attributed to their lower exfoliation degree and the stacking of 4–5 layers, which promotes sedimentation under gravity. With a zeta potential reaching ~+37 mV, the surface modification of GO was considered effective at an HDTMA concentration of 7.2 g L^−1^, and GO-HDTMA_14.6_ was selected for subsequent adsorption experiments. Prior to its use as an adsorbent matrix for pharmaceutical removal, GO-HDTMA_14.6_ was further characterized by Raman and FTIR spectroscopies, as well as thermogravimetric analysis. As expected, the Raman spectra of both GO and GO-HDTMA_14.6_ exhibit the two characteristic bands: the G band at ~1600 cm^−1^, associated with the graphitic structure, and the D band at ~1300 cm^−1^, related to the defects or induced by the presence of oxygen or other structural distortions in the graphitic structure [23,29,35]. These defects may arise from the small particle size of the precursor and its exposure under the laser beam. Indeed, Raman analysis performed on powder samples may involve particles oriented with exposed edges, leading to localized defect signals. For both GO samples, the I_D_/I_G_ ratio increases by approximately one order of magnitude to ~0.86, in agreement with previous studies, and remains unchanged after modification with the cationic surfactant (Figure 5b). This indicates that HDTMA adsorption does not significantly alter the sp^2^ carbon network, nor does it increase the defect density, supporting the absence of covalent bonding between HDTMA and GO [23,29,35].

Fourier transform infrared (FTIR) spectroscopy, performed using an ATR diamond crystal in this study, provides complementary insights into the material, particularly regarding the influence of the surfactant and its possible conformations and organization on the GO surface. The absence of characteristic bands associated with the sp^2^ carbon framework of graphite likely arises from the symmetry of C–C bonds, which are infrared inactive. In contrast, graphene oxide exhibits numerous absorption bands attributed to oxygen-containing functional groups grafted onto the graphitic surface, consistent with literature reports and confirming effective oxidation of graphite. A broad absorption band centered at ~3214 cm^−1^ is observed and can be assigned to O–H stretching vibrations, mainly due to adsorbed water (Figure 5a). An additional signal around 3600 cm^−1^ is attributed to hydroxyl groups of the oxidized graphitic structure, while the band at 1223 cm^−1^ corresponds to C–OH stretching vibrations. Carbonyl-containing groups, such as carboxylic acids (–COOH), also contribute to absorption features in this region, whereas epoxide groups (–C–O–C–) are identified by a band at lower wavenumber (~1048 cm^−1^). The C=C stretching mode of the sp^2^ carbon framework is observed at ~1621 cm^−1^. For GO-HDTMA, three additional absorption bands appear at 2919, 2849, and 1466 cm^−1^, which are assigned to the cationic surfactant [1,2,23,29,35]. These bands correspond to C–H stretching and bending modes of the long aliphatic chains of HDTMA. The persistence of the oxygen-containing functional group bands indicates that graphene oxide was neither reduced nor structurally altered upon surfactant adsorption (Figure 3d). Moreover, slight shifts in the wavenumber positions of oxygen-related bands (e.g., C–O–H) are observed after HDTMA adsorption. These variations may arise from interactions with the surfactant and/or band overlap. As a cationic species, HDTMA is expected to interact primarily through electrostatic (Coulombic) interactions with negatively charged oxygen functionalities of GO, particularly hydroxyl and carboxyl groups. Such interactions can locally modify the chemical environment of GO and thus affect the vibrational frequencies of these functional groups [23,29,35]. While FTIR analysis qualitatively confirms the presence of HDTMA on the GO surface, thermogravimetric analysis (TGA) provides a quantitative assessment of its loading. The surfactant content is estimated to be approximately 24 wt% of the total mass of the hybrid material, as evidenced by a mass loss in the temperature range of 200–400 °C, corresponding to the thermal degradation of the cationic surfactant (Figure 5c).

The adsorption of the three pharmaceuticals (PPs) was conducted at a constant pH of 5, adjusted by adding identical volumes of NaOH and HCl solutions in equivalent proportions, acting as buffered solutions, to all colloidal dispersions. This approach ensures consistent experimental conditions, including identical ionic strength and a well-defined pH at which the speciation of the pharmaceuticals is known (Figure 2). The concentrations of the pharmaceuticals before and after contact with the adsorbents were determined by UV–visible spectroscopy. As all compounds contain aromatic rings, they exhibit characteristic absorption bands in the UV–visible region, with maximum absorbance at 353, 222, and 268 nm for DCF, MTP, and SMX, respectively. A linear relationship between absorbance and concentration was observed over the ranges of 5–40, 5–70, and 5–15 mg L^−1^ for DCF, MTP, and SMX, respectively. This widely used method for evaluating adsorption efficiency relies on the Beer–Lambert law, which relates the absorbance of light in the UV–visible range to the concentration of a solute in a clear solution. The presence of colloidal particles, such as suspended adsorbents, may induce light scattering and interfere with absorbance measurements. Therefore, samples were centrifuged and filtered through a 0.22 μm membrane prior to analysis. The resulting supernatants exhibited no Tyndall effect, confirming effective removal of particulate matter. Furthermore, the linear dependence of absorbance on pharmaceutical concentration was verified in the supernatants after contact with GO or GO-HDTMA, within the experimental ranges of 0.5–2, 0.02–0.5, and 0.01–0.2 g L^−1^ for DCF, MTP, and SMX, respectively. Accordingly, this mass-balance approach, combined with accurate determination of contaminant concentrations before and after adsorption, enables reliable quantification of pharmaceutical uptake onto both GO and GO-HDTMA_14.6_ adsorbents using UV–visible spectroscopy [9,28,36].

While the Langmuir model was initially defined for the sorption of gas molecules to a solid surface, this model was extended for the description of the adsorption of solute molecules onto porous material. The Langmuir equation is expressed as follows (1):

(1) $$q_{e}=\frac{q_{max}K_{L}C_{e}}{1+K_{L}C_{e}},$$

where *q_e_* is the equilibrium pharmaceutical amount adsorbed on GO and GO-HDTMA_14.6_ (mmol g^−1^), *C_e_* the equilibrium concentration of the pharmaceutical in the resulting solution (mol L^−1^), *q_max_* is the maximum adsorption capacity of the sorbents (mmol g^−1^) and *K_L_* is the Langmuir adsorption constant (L mmol^−1^), determined within the experimental concentration range of the PP investigated here. The standard free energy (∆G°) of adsorption can be then calculated by the relation (2):

(2) $$∆G{}^{\circ}=-RT\ln\left(K_{L}C_{0}\right),$$

where $C_{0}$ corresponds to a standard reference concentration of 1 mM, appropriate for highly diluted solute concentrations and the behavior of its adsorption onto porous materials. Since GO shows a certain diversity in the surface landscape as shown by XPS data (Figure 3), and thus exhibits a heterogeneity in the distribution of the adsorption sites, the Freundlich model isotherm equations were used. The Freundlich isotherm is a semi-empirical equation to describe heterogeneous systems of which the linear form is (3):

(3) $$\ln q_{e}=lnK_{F}+\frac{1}{n}lnC_{e},$$

where *K_F_* (L g^−1^) and n are Freundlich adsorption isotherm constants, determined within the experimental concentration range, being indicative of the extent of the adsorption and the degree of nonlinearity between the solute concentration and adsorption, respectively. The standard Freundlich constant $K_{F}^{{}^{\circ}}$ (mmol g^−1^) can be expressed with the standard concentration *C*_0_ (1 mM) of a solute (pharmaceutical compound here) by the following Equation (4):

(4) $$K_{F}^{{}^{\circ}}=K_{F}C_{0}^{1/n}$$

**Figure 6:**
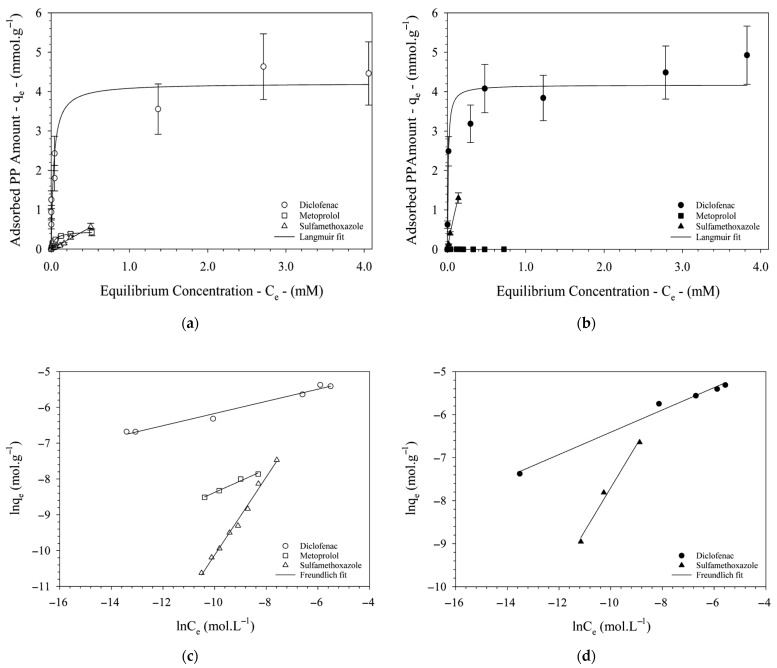
(**a**) Adsorption isotherms of diclofenac (circle), metoprolol (square), and sulfamethoxazole (triangle) onto both GOs with experimental data fitted by the Langmuir model (solid line). (**b**) Adsorption isotherms of the same pharmaceuticals onto GO-HDTMA_14.6_ with experimental data fitted by the Langmuir model. (**c**) Freundlich model fit of the adsorption data on GO and (**d**) Freundlich model fit of the adsorption data on GO-HDTMA_14.6_.

The adsorption isotherms of diclofenac (DCF) onto GO and GO-HDTMA_14.6_ exhibit a similar two-step behavior (Figure 6). Initially, the amount of adsorbed DCF increases with increasing solute concentration, followed by a plateau corresponding to the saturation of available adsorption sites. Although the Langmuir model provides a reasonable fit to the experimental data (Table 1), it does not appear to be the most appropriate model for describing DCF adsorption on either GO or GO-HDTMA_14.6_, as indicated by relatively moderate correlation coefficients (*r*^2^ ≈ 0.91). In contrast, the Freundlich model shows a significantly better agreement with the experimental data, with *r*^2^ values exceeding 0.99. Because the use of logarithmic scales may mask experimental deviations, an additional error function (*F_error_*) was employed to more accurately assess the quality of the model fitting. Lower *F_error_* values indicate a smaller discrepancy between the calculated adsorption capacity (*q_cal_*) and the experimental values (*q_exp_*). The error function is defined as: $F_{error}=\sum_{i}^{P}{\left(\frac{q_{i\;cal}-q_{i\;exp}}{q_{i\;exp}}\right)}^{2}$, where *q_ical_* is the adsorption capacity predicted by the model, *q_exp_* is the experimentally measured value, *i* refers to the different initial concentrations of the pharmaceutical, and P is the total number of experiments. The trends observed for *F_error_* are consistent with those of the correlation coefficients, further supporting the reliability of the fitting analysis. Both fitting error coefficients support the robustness of the Freundlich model in contrast to the Langmuir one. However, both models fit properly the experimental data that show here a saturation of the accessible surface area of the two adsorbents. The Langmuir model relies on the assumption of monolayer adsorption on a structurally homogenous adsorbent, whose sorption sites are all identical and energy-equivalent. In contrast, the Freundlich model is based on the adsorption on heterogeneous surfaces, where the interaction between the adsorbed molecules is not limited to the formation of a monolayer. Thus, due to the heterogeneity of the surface landscape of GO, whatever its lateral size, with multiple and various adsorption sites, it is not surprising that the Freundlich model appears to be the most appropriate model for fitting the experimental data, but the steady state of the adsorption of DCF supports the validity of the Langmuir model with the assumption of a homogeneous potential for the adsorption. The maximum adsorption capacities are comparable for both adsorbents, reaching approximately 4.2 mmol g^−1^. The negative values of Δ*G*°, determined by the Langmuir model, indicate that the adsorption process is spontaneous. The good affinity of DCF toward both carbon-based nanosheets is further confirmed by the Freundlich model. Moreover, the value of the heterogeneity parameter (*n* > 1) indicates favorable adsorption and suggests a heterogeneous distribution of adsorption sites, which is consistent with the heterogeneous surface landscape of both GO and GO-HDTMA_14.6_.

**Figure 7:**
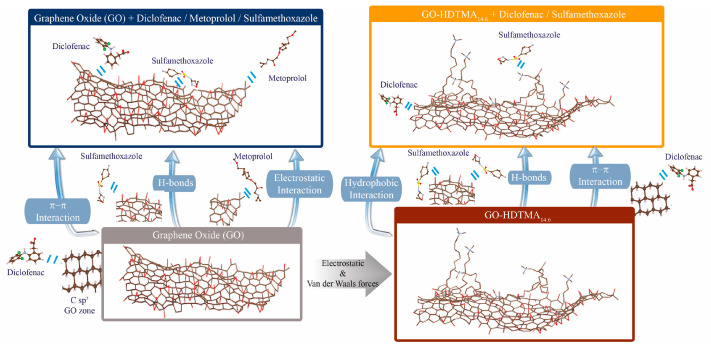
Conceptual representation of the adsorption mechanisms leading to the adsorption of diclofenac, metoprolol and sulfamethoxazole onto both anionic GO and cationic GO-HDTMA_14.6_.

The standard Freundlich constant $K_{F}^{{}^{\circ}}$, reflecting the capacity of an adsorbent to efficiently remove a given amount of contaminant, reached comparable values for both adsorbents. Nevertheless, the latter system was less predictable regarding the speciation of DCF and the overall surface nature of the carbonaceous nanosheets, despite the heterogeneity of their adsorption sites. Indeed, since DCF is predominantly present in its anionic form, one might expect an enhanced adsorption onto the GO-HDTMA_14.6_ hybrid material, with an increase in both the maximum adsorption capacity and $K_{F}^{{}^{\circ}}$ by one order of magnitude due to potential attractive electrostatic interactions favoring adsorption of the anti-inflammatory compound. However, the similar magnitudes of both $K_{F}^{{}^{\circ}}$ and the experimental maximum adsorption capacities suggest that electrostatic interactions are not the dominant mechanism governing DCF adsorption on the carbon surface. Both experimental studies and molecular simulations have previously demonstrated that DCF adsorption on graphene or graphite sheets is favorable and spontaneous, primarily driven by weak van der Waals and π–π interactions (Figure 7) [8,11,23]. In the case of GO, these weak interactions mainly occur between the sp^2^-hybridized carbon domains of GO and the aromatic or hydrophobic moieties of DCF, in addition to possible hydrogen bonding or ion–dipole interactions, albeit to a much lesser extent, between protonated DCF species and the graphitic nanosheets. All these interaction mechanisms lead to DCF adsorption on GO at a similar magnitude to that observed for its organo-modified analogue. In addition, the HDTMA coating not only modifies the zeta potential but also enhances surface hydrophobicity, promoting interaction between the hydrophobic moieties of DCF and HDTMA. This can be emphasized if one normalizes the adsorbed amount of DCF by the real active mass of GO involved in the adsorption. For GO-HDTMA, the inorganic nanosheets represent 75 wt% of the total mass of the hybrid material. Thus, based on this assumption, GO-HDTMA may represent an efficient material for the removal of DCF with an enhancement of the adsorption of 25% (corresponding to the increase of mass due to the grafting or association of HDTMA), that can be possible due to the hydrophobic environment generated by the long aliphatic chains of HDTMA. Nevertheless, in both adsorbents, the sp^2^ carbon zones are the main sites responsible for the adsorption of DCF. These graphitic zones represent a uniform area covered with equivalent adsorption sites for which the Langmuir assumption can be applied.

The estimated molecular radius of DCF is approximately 0.5 nm, corresponding to an apparent packing area of ~0.75 nm^2^ [11]. Assuming a theoretical specific surface area of ~2000 m^2^ g^−1^ for both GO and GO-HDTMA_14.6_, the accessible graphitic carbon surface can be estimated at ~792 m^2^ g^−1^ when accounting for the oxidation degree of the carbonaceous nanosheets determined by XPS (Figure 3) [37]. Considering this sp^2^ carbon surface and assuming adsorption of isolated DCF molecules, the maximum number of DCF molecules covering the accessible graphitic surface is calculated to be ~17.2 mmol g^−1^ [23,38]. Based on the assumption of the full accessibility of this hydrophobic surface and complete exfoliation of the nanosheets, the experimentally determined adsorbed amounts correspond to only ~25% of the theoretically available graphitic surface of GO [39]. However, the adsorption isotherms of DCF onto both GO and GO-HDTMA_14.6_ exhibit a plateau at high concentrations, suggesting saturation of the sp^2^ carbon surface. Notably, this plateau remains similar for both adsorbents, indicating that chemical modification does not significantly alter the number of accessible graphitic sites. These observations underline a limitation of both adsorbents for DCF removal, arising from saturation of the available graphitic domains. Furthermore, AFM images support partial exfoliation of the carbon nanosheets, revealing aggregates composed of 4–5 stacked GO layers with an overall thickness of ~5 nm. The exfoliation ratio can also be estimated by considering the experimental adsorption capacity as the maximum achievable value, yielding ~25%, consistent with the previous calculation. Thus, GO and its modified analogue are likely dispersed as multilayer particles or aggregates comprising approximately 3–5 nanosheets, which restricts the accessible graphitic surface proportionally to the stacking degree [16,17,39]. Adsorption studies using small probe molecules such as DCF therefore provide a useful approach to estimate the exfoliation ratio of GO, in agreement with AFM observations (Figure 3). Despite the limitations associated with incomplete exfoliation, the adsorption capacity of GO for DCF obtained in this study (4.2 mmol g^−1^) exceeds previously reported values (~0.405 mmol g^−1^) for activated carbons or (~0.432 mmol g^−1^) for GO and (~0.105 mmol g^−1^) for other composite adsorbents based on GO by nearly one order of magnitude (see Table 2 and Figure S3 for an enlarged view of the adsorption isotherms as well as Figure S4 for a comparison in mg per gram of adsorbent), highlighting the versatility and strong potential of GO and its organo-modified analogue as advanced adsorbent materials [8,40,41].

While surface charge appears to play a minor role in the adsorption of diclofenac (DCF) onto GO and GO-HDTMA_14.6_, it becomes a key determining factor for metoprolol (MTP), a cationic compound, for which no adsorption onto GO-HDTMA_14.6_ was observed (Figure 6 and Table 1). Repulsive electrostatic interactions between the positively charged HDTMA-coated surface and MTP hinder adsorption, despite the hydrophobic character of the analyte. In contrast, although MTP exhibits limited solubility in water, its adsorption onto pristine GO occurs spontaneously and displays a two-stage isotherm similar to that observed for DCF: a linear increase at low concentrations followed by a plateau at higher concentrations. This plateau indicates saturation of negatively charged oxygen-containing functional groups on GO (e.g., hydroxyl and carboxyl groups), leading to a maximum adsorption capacity of 0.5 mmol g^−1^, consistent with previously reported values for similar adsorbent systems but sensitively lower to that (~1.67 mmol g^−1^) of the composite based on magnetite particles, where adsorption is also governed by electrostatic interactions [42,43]. The negative Gibbs free energy confirms the spontaneous nature of the process, while its magnitude supports the involvement of attractive electrostatic interactions. In addition, the Freundlich constant (*n* > 1) indicates favorable adsorption. Nevertheless, the adsorption capacity of GO toward MTP remains limited, with a Freundlich constant approximately one order of magnitude lower than that of DCF. This difference highlights the predominant role of the hydrophobic sp^2^ carbon surface of the nanosheets in the adsorption of lipophilic compounds. Here again, the saturation of the steady state of the adsorption indicates the full covering of the adsorption accessible sites of GO for MTP, with a line shape supporting the validity of the Langmuir model. Electrostatic attractive force is the main interaction mechanism leading to the adsorption of MTP onto GO, for which all negatively charged carboxyl groups are covered by the beta-blocker pharmaceutical in monolayer through the same homogenous potential.

Lastly, sulfamethoxazole (SMX), which is predominantly nonionic at the studied pH, exhibits weak adsorption onto GO, as evidenced by low uptake and the absence of a plateau in the adsorption isotherm. The Freundlich model yields *n* ≈ 1, indicating borderline or unfavorable adsorption behavior, consistent with previous reports for similar adsorbents [45]. A comparable trend is observed for SMX adsorption onto GO-HDTMA_14.6_, albeit with a moderately increased adsorption capacity. This enhancement can be attributed to the hydrophobic microenvironment created by the long alkyl chains of HDTMA, as well as possible ion–dipole interactions between residual cationic surfactant groups and SMX. These results suggest that chemical modification of GO with HDTMA yields a more versatile adsorbent capable of capturing both hydrophobic and nonionic species, with improved uptake relative to pristine GO. Interestingly, both $K_{F}^{{}^{\circ}}$ and the maximum adsorption capacity estimated for GO-HDTMA_14.6_ toward SMX appear relatively high. However, these values should be interpreted with caution, as they are derived from a limited concentration range constrained by the low solubility of SMX. Consequently, the available dataset does not provide sufficient resolution to ensure high accuracy in the fitting of experimental data, despite an apparent agreement with both Langmuir and Freundlich models. Therefore, the fitting parameters obtained from these models should be considered with care. Moreover, with a value of *n* (determined by the Freundlich firring procedure) close to 1, this also supports adsorption of SMX following a Langmuir adsorption model despite the heterogeneity of the adsorbents and speciation of SMX as well as its hydrophilic behavior. In such cases, direct comparison based on experimentally measured adsorption capacities offers a more reliable approach for evaluating adsorbent performance. The experimentally determined adsorption capacity of SMX onto GO-HDTMA_14.6_ reaches 1.3 mmol g^−1^, which is slightly higher than values reported for comparable materials, indicating a moderate improvement in adsorption efficiency [44].

**Figure 8:**
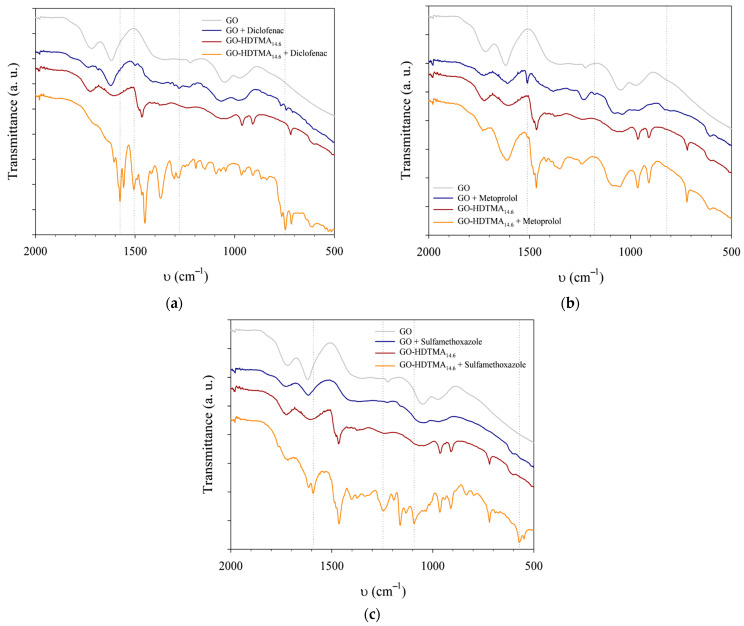
(**a**) Fourier transform infrared spectra of GO and GO-HDTMA before and after being in contact with that diclofenac; (**b**) same with metoprolol; (**c**) same with sulfamethoxazole.

Infrared spectroscopy of the collected solid residues (Figure 8) further confirms the adsorption of the pharmaceutical compounds onto both GO and GO-HDTMA materials. Characteristic absorption bands of DCF are observed near 750 cm^−1^, corresponding to stretching vibrations associated with C–Cl bonds on the aromatic rings. Other characteristic DCF bands are not clearly resolved due to overlap with GO and HDTMA absorption features. In addition, adsorption onto solid surfaces may significantly alter the molecular environment, thereby affecting the spectroscopic response of the adsorbed species. In this context, DCF adsorption follows a Langmuir-type behavior, consistent with monolayer coverage of the GO surface without lateral interactions between adsorbed molecules, as supported by infrared spectroscopy [2,28,36].

Metoprolol signals are detected only in GO samples, with a band at 1510 cm^−1^ characteristic of aromatic C–C stretching vibrations. This observation is consistent with UV–visible spectroscopy results and confirms the preferential adsorption of the cationic compound onto the negatively charged GO surface. Because only GO solids recovered after exposure to high MTP loadings were analyzed, it was not possible to assess potential conformational changes of adsorbed MTP molecules or to correlate the evolution of band intensity with MTP concentration in a manner comparable to the adsorption isotherm [2,9].

SMX exhibits a weaker infrared response, consistent with its low adsorption capacity, which arises from its limited solubility in water and potential overlap with GO and HDTMA vibrational bands. Its adsorption is nevertheless suggested by a weak absorption feature between 790 and 830 cm^−1^, attributed to deformation modes of aromatic rings, which is only detectable for GO-HDTMA_14.6_ nanosheets. Collectively, these spectroscopic features confirm the successful adsorption of the investigated pharmaceutical compounds onto both GO-based adsorbent materials.

## 4. Conclusions

This study demonstrates the successful synthesis and functionalization of graphene oxide (GO) nanosheets with the cationic surfactant hexadecyltrimethylammonium (HDTMA), yielding a versatile hybrid material with tunable surface charge and adsorption properties. Structural, morphological, and spectroscopic characterizations confirm the efficient immobilization of HDTMA onto the GO surface, resulting in a positively charged composite and the introduction of additional adsorption sites. This modification enhances the capacity of the material for the removal of organic contaminants with pronounced hydrophobic character. Adsorption isotherm experiments reveal distinct affinities of the hybrid material toward pharmaceutical compounds with contrasting physicochemical properties, including hydrophobicity, charge, and therapeutic function. Diclofenac (DCF), present predominantly in its anionic form (~87%) with a minor neutral fraction (~13%), exhibits strong adsorption onto both pristine GO and GO-HDTMA_14.6_. This behavior is primarily governed by weak intermolecular interactions, including π–π interaction and dispersive forces, as previously reported for graphene and graphite systems. The adsorption capacity reaches 4.2 mmol g^−1^ for both GO and GO-HDTMA_14.6_, which is approximately one order of magnitude higher than values reported for activated carbons (~0.4 mmol g^−1^). In contrast, metoprolol (MTP), a cationic β-blocker, is preferentially adsorbed onto pristine GO via attractive electrostatic interactions, yielding adsorption capacities consistent with literature values for similar adsorbents [42]. No adsorption is observed on GO-HDTMA_14.6_ due to repulsive electrostatic interactions arising from the positively charged surface. Finally, sulfamethoxazole (SMX), a predominantly nonionic antibiotic under the studied conditions, exhibits limited adsorption onto GO but shows improved uptake on GO-HDTMA_14.6_. This enhancement is attributed to the hydrophobic environment generated by the HDTMA coating, which promotes interactions with nonpolar moieties of SMX. Overall, these results highlight the potential of GO and its organo-modified analogue to be efficient and versatile adsorbents for the removal of a wide range of pharmaceutical contaminants from aqueous environments. Future work should focus on optimizing surfactant loading and investigating regeneration and reuse cycles to improve the sustainability and scalability of these hybrid materials for water treatment applications.

## Figures and Tables

**Table 1:** Adsorption isotherm constants determined with Langmuir and Freundlich model fits for the adsorption of the whole pharmaceutical products (PPs): diclofenac (DCF), metoprolol (MTP), and sulfamethoxazole (SMX) onto both GO and GO-HDTMA_14.6_.

Adsorbent	PP		Langmuir					Freundlich		
		*q_max_* (mmol g^−1^)	*K_L_* (L mmol^−1^)	∆G° (kJ mol^−1^)	*r^2^*	*F_error_*	$K_{F}^{{}^{\circ}}$ (mmol g^−1^)	*1/n*	*r^2^*	*F_error_*
GO	DCF	4.2	31.7	−8.56	0.9154	0.257	3.52	0.17	0.990	0.0032
	MTP	0.5	22	−7.65	0.9970	0.072	0.632	0.32	0.879	0.0124
	SMX	-	-	-	-	-	1.16	1.09	0.995	0.0032
GO-HDTMA_14.6_	DCF	4.2	104	−11.50	0.9658	0.128	3.93	0.25	0.992	0.0042
	MTP	-	-	-	-	-	-	-	-	-
	SMX	6.3	2	−1.17	0.999	0.045	7.07	1.05	0.993	0.0028

**Table 2:** Comparison of the maximum adsorbed capacities of DCF, MTP and SMX for the studied GO and GO-HDTMA_14.6_ to those of previous materials based on GO.

Pharmaceuticals	Adsorption Materials	Adsorption Capacities (mmol g^−1^)	References
Diclofenac (DCF)	Activated carbon	0.405	[8]
	GO	0.432	[40]
	GO@CoFe_2_O_4_	~0.105	[41]
	GO (this study)	4.2	-
	GO-HDTMA (this study)	4.2	-
Metoprolol (MTP)	GO	~0.5	[42]
	Fe_3_O_4_@SiO_2_/SiCRG	~1.67	[43]
	GO (this study)	0.5	-
Sulfamethoxazole (SMX)	GO	0.92	[44]
	GO-HDTMA (this study)	~0.13	-

## Data Availability

The original contributions presented in this study are included in the article/Supplementary Materials. Further inquiries can be directed to the corresponding authors.

## Supplementary Materials

The following supporting information can be downloaded at: https://www.mdpi.com/article/10.3390/ma19101916/s1, Figure S1: TEM observations of graphene oxide at different magnifications; Figure S2: AFM observation of graphene oxide at a large magnification; Figure S3: (a) Enlarged view of the adsorption isotherms of diclofenac (circle), metoprolol (square), and sulfamethoxazole (triangle) onto both GO with experimental data fitted by the Langmuir model (solid line) and (b) enlarged view of the adsorption isotherms of the same pharmaceuticals onto GO-HDTMA_14.6_ with experimental data fitted by the Langmuir model; Figure S4: (a) Adsorption isotherms of diclofenac (circle), metoprolol (square), and sulfamethoxazole (triangle) onto both GO with experimental data fitted by the Langmuir model (solid line) and (b) adsorption isotherms of the same pharmaceuticals onto GO-HDTMA_14.6_ with experimental data fitted by the Langmuir model; Table S1: Detail of the deconvolution of the XPS spectrum of graphene oxide deposited on a Si substrate.

## Abbreviations

The following abbreviations are used in this manuscript:

PPs	Pharmaceutical products
DCF	Diclofenac
MTP	Metoprolol
SMX	Sulfamethoxazole
GO	Graphene oxide
HDTMA	Hexadecytrimethylammonium
