# Supplementary material for: Versatile Graphene Oxide and Its Organo-Modified Analogs for the Removal of Pharmaceutical Compounds
Source: Materials (Basel). 2026 May 7;19(10):1916. doi: 10.3390/ma19101916 (PMC13209116; doi:10.3390/ma19101916)
Supplement: Supplementary file 1 [file materials-19-01916-s001.zip › materials-4266491-supplementary.pdf]

Article

Supplementary information: Versatile Graphene Oxide and Its Organo-Modified Analogs for the Removal of Pharmaceutical Compounds

Emilie Fragnaud [1,2], Louis Hennet [2,*], Eric Bourhis [2], Samuel Guillot [2], Sandrine Delpeux [2], Fabrice Muller [2], Yoshiyuki Sugahara [3] and Régis Guégan [2,*]

[1] Institut des Sciences Chimiques de Rennes, ISC-UMR 6226, CNRS-Université de Rennes, 263 Avenue du Général Leclerc, 35700 Rennes, France; emilie.fragnaud@univ-rennes.fr

[2] Interfaces, Confinement, Matériaux et Nanostructures, ICMN-UMR 7374, CNRS-Université d'Orléans, 3 Avenue de la Recherche Scientifique, 45071 Orléans CEDEX 2, France; eric.bourhis@cnrs-orleans.fr (E.B.); samuel.guillot@cnrs-orleans.fr (S.G.); sandrine.delpeux@cnrs-orleans.fr (S.D.); fabrice.muller@univ-orleans.fr (F.M.)

[3] Department of Applied Chemistry, Faculty of Science and Engineering, Waseda University, 3-4-1 Okubo, Shinjuku-ku, Tokyo 169-8555, Japan

* Correspondence: louis.hennet@cnrs-orleans.fr (L.H.); regis.guegan@univ-orleans.fr (R.G.); Tel.: +33-(0)2-38-25-53-57 (L.H.); +33-(0)2-38-25-53-18 (R.G.)

Table S1. Detail of the deconvolution of the XPS spectrum of graphene oxide deposited on a Si substrate.

C-O-C/C-OH	C=O	C=C/C-C	C-H
57.55%	5.5%	13.5%	23.45%

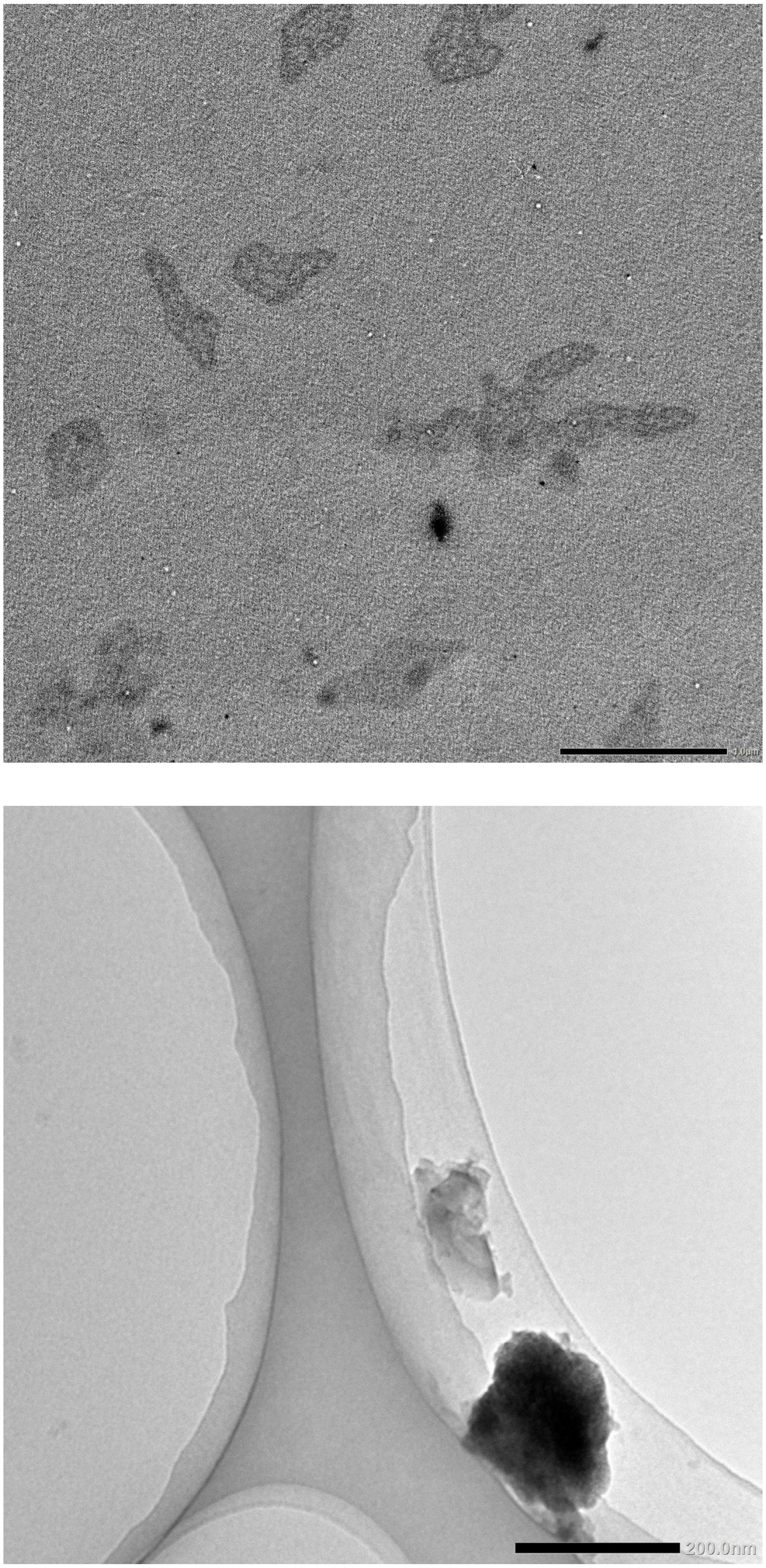

Figure S1. (a & b) TEM observations of graphene oxide at different magnifications.

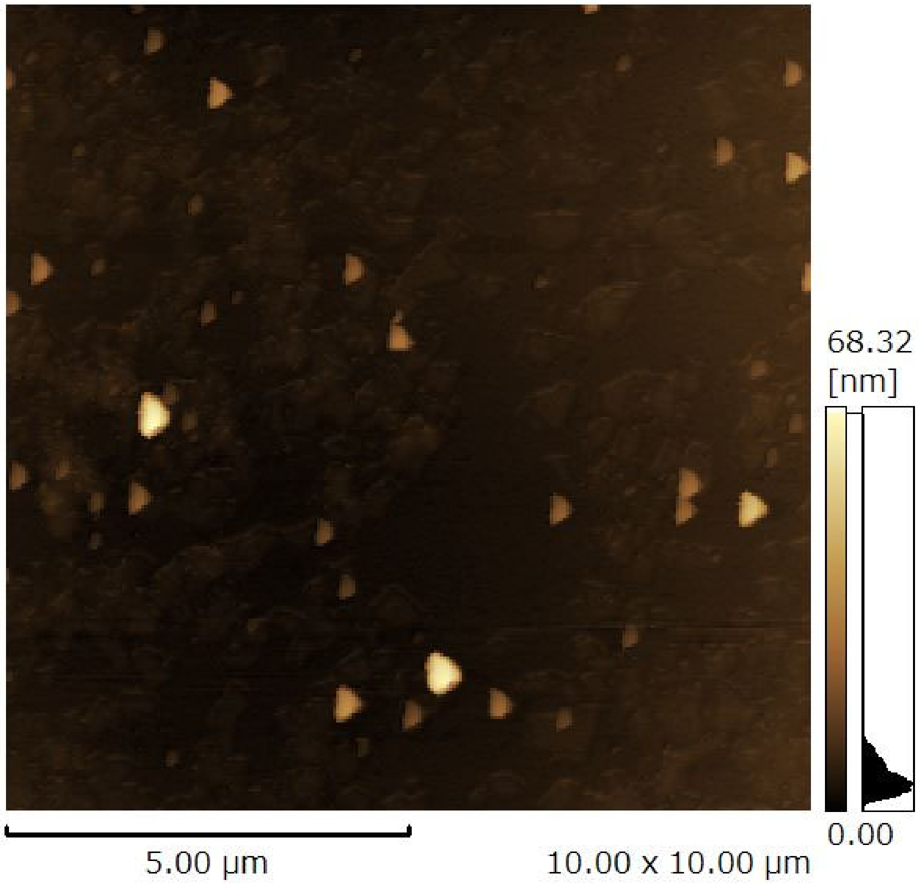

Figure S2. AFM observation of graphene oxide at a large magnification.

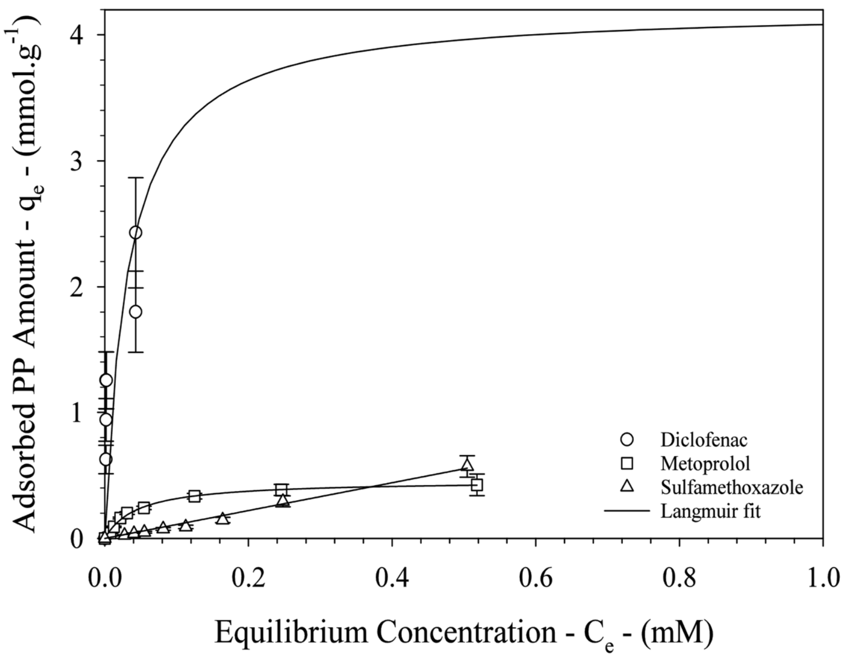

(a)

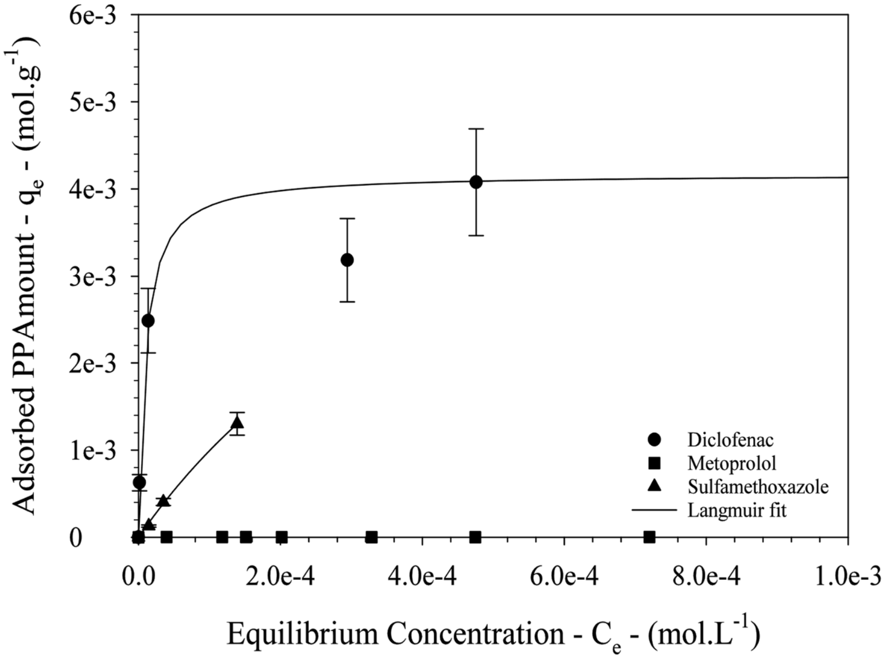

(b)

Figure S3. (a) Enlarged view of the adsorption isotherms of Diclofenac (circle), Metoprolol (square), and Sulfamethoxazole (triangle) onto both GO with experimental data fitted by Langmuir model (solid line) (b) Enlarged view of the adsorption isotherms of the same pharmaceuticals onto GO-$HDTMA_{14.6}$ with experimental data fitted by a Langmuir model.

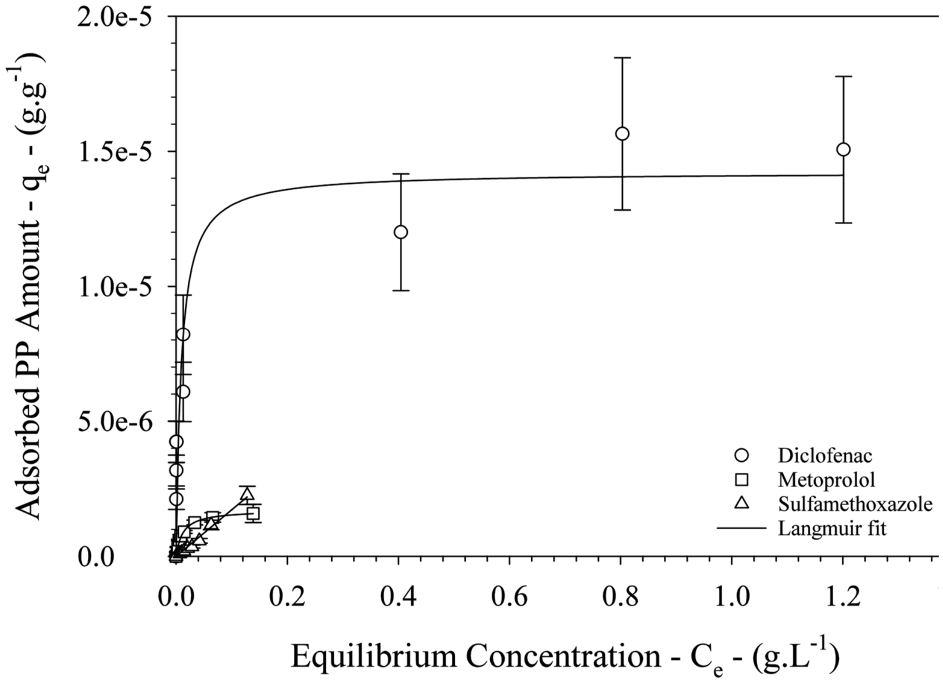

(a)

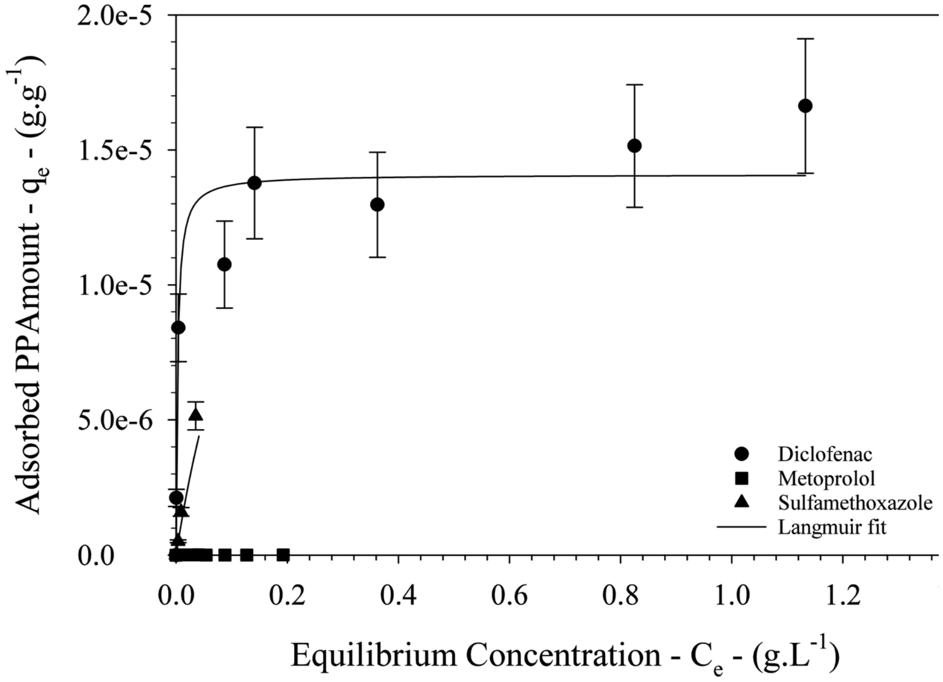

(b)

Figure S4. (a) Adsorption isotherms of Diclofenac (circle), Metoprolol (square), and Sulfamethoxazole (triangle) onto both GO with experimental data fitted by Langmuir model (solid line) (b) Adsorption isotherms of the same pharmaceuticals onto GO-$HDTMA_{14.6}$ with experimental data fitted by a Langmuir model.
